# The prognostic value of mean platelet volume levels in germinal matrix hemorrhage- intraventricular hemorrhage in preterm infants

**DOI:** 10.12669/pjms.41.4.11642

**Published:** 2025-04

**Authors:** Ipek Guney Varal, Ezgi Deniz Acar Celik, Pelin Dogan, Gaffari Tunc, Ayse Oren

**Affiliations:** 1 Ipek Guney Varal Division of Neonatology, Department of Paediatrics, University of Health Sciences, Bursa Yuksek Ihtisas Teaching Hospital, Bursa, Turkey; 2 Ezgi Deniz Acar Celik Department of Paediatrics, University of Health Sciences, Bursa Yuksek Ihtisas Teaching Hospital, Bursa, Turkey; 3 Pelin Dogan Division of Neonatology, Department of Paediatrics, University of Health Sciences, Bursa Yuksek Ihtisas Teaching Hospital, Bursa, Turkey; 4 Gaffari Tunc Division of Neonatology, Department of Paediatrics, University of Health Sciences, Bursa Yuksek Ihtisas Teaching Hospital, Bursa, Turkey; 5 Ayse Oren Division of Neonatology, Department of Paediatrics, University of Health Sciences, Bursa Yuksek Ihtisas Teaching Hospital, Bursa, Turkey

**Keywords:** Germinal matrix, Intraventricular hemorrhage, Mean platelet volume, Neonate, Preterm

## Abstract

**Objective:**

Germinal matrix hemorrhage and intraventricular hemorrhage (GMH-IVH) is currently the most significant cause of brain damage and mortality seen in preterm infants. The aim of this study was to evaluate the predictive value of mean platelet volume (MPV) in diagnosing GMH-IVH and mortality.

**Method:**

A retrospective-cohort study of preterm infants with a gestational age <32 weeks or with birthweight <1500gr who were admitted to the Neonatal Intensive Care Unit was conducted between January 2020 and January 2022. These infants were then classified into two groups according to the presence of GMH-IVH.

**Results:**

Overall, 136 preterm infants were enrolled. The MPV levels were significantly higher in the GMH-IVH group (p <0.001). A MPV cutoff of >9.95 was determined to be predictive for GMH-IVH with sensitivity of 62% and specificity of 80% (p < 0.001). Mortality was significantly higher in the GMH-IVH group (p<0.001). A MPV cutoff of >10.55 fL was determined to predict mortality with 77% sensitivity and 70% specificity (p < 0.001).

**Conclusions:**

The MPV value is significantly higher in infants with GMH-IVH and therefore can be used in the prediction of GMH-IVH and mortality in preterm infants.

## INTRODUCTION

Germinal matrix hemorrhage and intraventricular hemorrhage (GMH-IVH) is the most significant cause of brain damage and mortality seen especially in preterm infants. The frequency is in inverse proportion to maturity, and preterm infants born before 32 gestational age (GA) and <1500 gr are at greater risk. Approximately 45% of the very small preterm group, born before 26 GA, are affected by GMH-IVH.^1^ In the brain vascularisation of preterms, the germinal matrix is a special vascular structure with increased permeability when there is an increase in hypoxia and venous pressure, and this structure cannot be classified as arteriole, venule, or capillary vesssels. This structure, which continues until the 36^th^ GA, lays the ground for hemorrhage to occur more easily.^2^ The risk of GMH-IVH is higher especially in the first days of life and preterms are monitored at regular intervals with cranial ultrasonography. Several different conditions can change in cerebral blood flow can lead to severe hemorrhage, which can then result in lifelong disabilities. To prolong life in preterms, it is crucial to aim for a reduction in GMH-IVH, to prevent it and to identify risk groups.

Mean platelet volume (MPV) reflects the mean thrombocyte size and is determined using hematology analyzers. Thrombocyte volume is determined during the formation of thrombocytes from megakaryocytes, and therefore, conditions stimulating bone marrow can cause changes in thrombocyte number and volume. When bone marrow is stimulated, changes in thrombocyte production and thrombocyte maturation cause an increase in MPV. 3 Young thrombocytes are larger than older thrombocytes. There is a greater density of granules in large volume platelets compared to small thrombocytes and more serotonin and β-thromboglobulin is expressed. At the same time, more young thrombocytes show greater production and consumption.^4^

The ratio of mean thrombocyte volume to thrombocyte count, mean platelet ratio (MPR), which is calculated by dividing the mean platelet volume by the platelet count, is a simple marker of bone marrow and megakaryocytes. MPV also shows endothelial damage and platelet activation.^3^ In view of this knowledge, the hypothesis of this study was that platelet parameters, which are critical for endothelial damage, would be associated with GMH-IVH and mortality in preterm infants.

## METHODS

A retrospective-cohort study of preterm infants with a gestational age <32 weeks or with birthweight <1500gr who were admitted to the Neonatal Intensive Care Unit was conducted between January 2020 and January 2022.

### Ethical Approval:

Ethics committee approval was granted by the local ethics committee of the university hospital (2024-TBEK 2024/12-10, dated January 31, 2024).

These infants were then classified into two groups according to the presence of GMH-IVH. All the patients were evaluated by the same two neonatologists according to the defined preterm ultrasonographic monitoring protocol.^1^ Cranial ultrasound scanning (cUS) was performed in all preterm once or twice within the first 72 hours after first admission, on average on day three, and weekly thereafter. Classification of GM-IVH was made as described by Volpe.^2^ Periventricular hemorrhagic infarction (PVHI) is defined as parenchymal hemorrhage on the ipsilateral side. For this study, Grade-I and Grade-II hemorrhages were classified as low-grade, and Grade-III and PVHI hemorrhages as high-grade.^5^

### Exclusion criteria:

Family history of hematological diseases, infants born to mothers with thrombocytopenia, use of drugs that may cause changes in blood cell morphology, infants with major congenital and chromosomal anomalies, and incomplete case data.

A record was made for each case of fetal and antenatal demographic data with mortality. Blood samples were obtained from all the preterms during the first 24 hours of life on admission to the NICU. The hematological parameters were determined using a Sysmex-XT-2000i counter (Sysmex, Kobe, Japan). The complete blood-count parameters including MPV were recorded. MPR was calculated as the ratio of MPV and platelet count. The MPV and MPR values were compared within and between the groups.

### Statistical analyses:

The data obtained in the study were analysed statistically using SPSS version 22.0 software (SPSS Inc, Chicago, IL, USA). Chi-square analysis or Fisher’s Exact test was used to compare categorical variables between the groups. The Mann–Whitney U-test and Kruskal Wallis tests were applied to compare non-parametric variables and the Wilcoxon-signed rank test was used in the comparisons of paired data. The analysis was presented with odds ratio (OR) and 95% confidence interval (CI) and the level of statistical significance was set at p <0.05. The receiver operating characteristics (ROC) curves were plotted for MPV and MPR, and cutoff values were determined.

## RESULTS

Throughout the defined study period, 3160 preterms were born in our hospital, of which 286 were admitted to the Neonatal Intensive Care Unit (NICU) as they were born at <32 GA. After the exclusion of 92 preterm infants due to incomplete data or not meeting the study inclusion criteria, a total of 194 preterms born at <32 GA were included in the study. According to the preterm ultasonographic monitoring protocol, GMH-IVH was determined in 58 (29%) infants (Group-I) and no intracranial hemorrhage was observed in 136 (71%) (Group-II). In the subgroup analyses of the study group, low-grade hemorrhage was determined in 44 (75%) preterms, and high-grade hemorrhage in 14 (25%). When the neonatal and maternal characteristics of the groups were compared, the birthweight and GA at birth were determined to be significaantly lower in Group-I than in Group-II (p: 0.05 and p: 0.06).

The demographic characteristics of the study populations are presented in Table-I. In the comparisons of the laboratory test results of the groups, the hemoglobin and hematocrit values of Group-I were determined to be statistically significantly lower than those of Group-II (p: 0.005). The MPV levels of Group-I were significantly higher than those of Group-II (median:10.7 fL, interquartile range (IQR): 7.6–14.6), (median: 9.9 fL, (IQR): 7.5-13.7) (p <0.001) (Table-II). No correlation was found between the MPV and MPR values and gestational age in the GMH-IVH group (p>0.05). When the GMH-IVH group was separated into subgroups of different gestational ages, no correlation was observed between the MPV and MPR values and birthweight (p>0.05).

**Table-I:** Neonatal and maternal characteristics of the study groups.

	Group-I (n=58)	Group-II (n=136)	P
Gestational age, week median (IQR)	25 (24-27)	29 (27-31)	0.006^a^
Birth weight, g median (IQR)	760 (620-850)	1130 (900-1390)	0.005^a^
*Sex, n (%)*			
Male	29 (50)	74 (54)	0.5^b^
Female	29 (50)	62 (46)	
Caesarean section, n (%)	37 (64)	83 (61)	0.4^b^
*Apgar score, median (IQR)*			
Minute 1	6 (4-6)	7 (5-8)	
Minute 5	7 (5-8)	8 (6-9)	0.06^a^
*Antenatal steroids, n(%)*			0.1^a^
No	34 (59)	69 (51)	
Single course	14 (24)	24 (17)	
Repeat course	10 (17)	43 (32)	
Preeclampsia, n (%)	12 (20)	23 (17)	0.2^b^
Chorioamnionitis, n (%)	3 (5)	8 (6)	0.2^b^
Multiparity, n (%)	38 (66)	76(56)	0.1^b^
Intrauterin growth restriction, n(%)	9 (15)	26 (19)	0.5^b^
Mortality, n(%)	22 (38)	30 (22)	<0.001^b^

a Mann-Whitney U test,

b Chi-square test IQR: interquartile range.

**Table-II:** Laboratory findings of the study groups.

	Group-I (n=58)	Group-II (n=136)	P
Prothrombin Time (PT), sn	13 (12.2-13.8)	14 (13-15)	0.2^a^
Activated Partial Thromboplastin Time (aPTT), sn	32 (28-35)	35 (27-36)	0.4^a^
International normalized ratio (INR)	1 (1-1)	1 (1-1)	0.5^a^
WBC count (10^3^/μl) median (IQR)	9.7 (4.4-11.6)	9.9 (7.1-11.7)	0.8^a^
Neutrophil count (10^3^/μl) median (IQR)	3.3 (2.9-5.6)	2.8 (2.5-4.6)	0.2^a^
Lymphocyte count (10^3^/μl) median (IQR)	3.8 (2.9-5.2)	4.4 (3.4-5.3)	0.1^a^
Hemoglobin (g/dl) median (IQR)	15 (14-17)	17 (16-19)	0.005^a^
Hematocrit (%) median (IQR)	45 (42-47)	51 (50-54)	0.005^a^
Platelet count (10^3^/μl) median (IQR)	215 (158-220)	211 (157-255)	0.4^a^
MPV (fl) median (IQR)	10.7 (7.6-14.6)	9.9 (7.5-13.7)	<0.001^a^
MPV/Platelet ratio (MPr) median (IQR)	0.07 (0.05-0.08)	0.06 (0.05-0.07)	0.4^a^

a Mann-Whitney U test, WBC: white blood cell; IQR: interquartile range; Sd: standart deviation; MPV: mean platelet volume; MPV/Platelet ratio (MPr)

**Fig.1:**
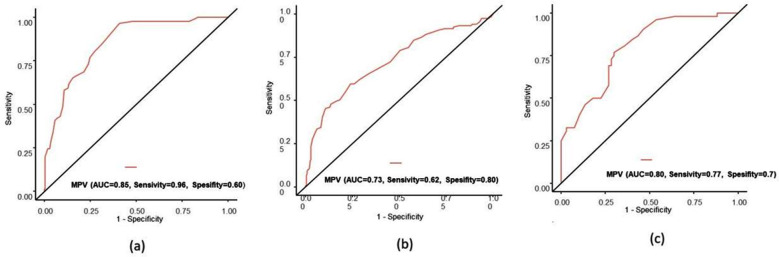
Receiver operating characteristics (ROC) (a) for predicting mortality and (b) for predicting GMH-IVH and (c) mortality in GMH-IVH preterms group.

The GMH-IVH group was separated into two subgroups of low-grade and high-grade hemorrhage, and there was seen to be no statistical significance between the demographic characteristics and laboratory results, with the exception of hemoglobin and hematocrit (p>0.05). The hemoglobin and hematocrit values were determined to be statistically significantly low in the low-grade hemorrhage group (p<0.001). When MPV value was compared in low grade and high grade haemorrhages, there was no statistical significance although it was higher in high grade haemorrhage (MPV values are 10.5fL, 10.9Fl, p:0.4, respectively). There was no statistical difference between the subgroups in coagulation test results, platelet and MPR values (p>0.05). Receiver operating characteristic (ROC) analysis was performed to determine the optimal threshold values of MPV for predicting GMH-IVH, and the area under the curve (AUC) was determined to be 0.73. An MPV value of 9.95 fL was found to have 62% sensitivity and 80% specificity (p < 0.001). ROC analysis was performed to determine the optimal threshold values of MPV for predicting mortality, and AUC was determined to be 0.85. The sensitivity and specificity values of an MPV value of 10.35fL were found to be 96% and 60%, respectively (p<0.001). ROC analysis was performed to determine the optimal threshold values of MPV for predicting mortality in the GMH-IVH group, and AUC was determined to be 0.80. An MPV value of 10.55fL was found to have 77% sensitivity and 70% specificity (p< 0.001) (Fig.1).

## DISCUSSION

Germinal matrix hemorrhage and intraventricular hemorrhage (GMH-IVH) is the most important cause of brain damage, which is seen especially in preterm infants, and despite recent developments in neonatal care, it continues to be a serious problem with negative effects on neurological development and mortality.^1^ The results of this study showed higher MPV values in blood samples taken in the first 24 hours from preterms determined with GMH-IVH. To the best of our knowledge, this is the one of the few studies in the literature to have evaluated the possible association between MPV and GMH-IVH and mortality together in preterm infants.

Although there are many risk factors for GMH-IVH in preterm infants, the most important causes are preterm birth and low birthweight.^5^,^6^ We demonstrated that the GA and birthweight of the preterms determined with GMH-IVH were significantly low. In our study included the highest risk group admitted to NICU, which was the preterms born <32 GA and <1500 gr, and the general frequency of GMH-IVH was seen to be 29%. Of the preterm infants determined with GMH-IVH, 76% were classified as low-grade, giving an incidence of high-grade hemorrhage of 10% of the whole study group. In current literature, the incidence of GMH-IVH has been reported to be 20-25%. It has also been reported that approximately 45% of the very small preterm group born before 26 GA are affected by GMH-IVH.^7^ Together with recent developments in neonatal care, a decrease has been seen in the rates of high-Grade-IVH, and it has been reported to be seen in 5-10% of preterms born at <32 GA.^8^

In this study, no relationship was determined between the frequency of GMH-IVH and the APGAR score, pre-eclampsia, chorioamnionitis, or intrauterine growth restriction. Although immaturity is the factor held most responsible for GMH-IVH, another important reason is perinatal asphyxia. Pre-eclampsia, which can lead to intrauterine hypoxia, chorioamnionitis and intrauterine growth retardation resulting from these have been associated with perinatal intracranial hemorrhage and other morbidities 9,10 However, just as antenatal steroid administration has been shown to reduce many complications in prematurity, it also reduces intracranial hemorrhages. In the current study, although antenatal steroid administration was greater in the group without GMH-IVH, no statistical significance was determined.

In our study, mortality in the GMH-IVH group was determined at a significantly high rate, at approximately 38%. In a recent large series, mortality rates were reported to be 4% for Grade-I and 10% for Grade-II. Mortality in the patients with low-grade hemorrhage generally occurred because of other underlying diseases, and mortality in high-grade hemorrhages was at the rate of 40%.^2^ Mortality is closely associated to GA, comorbidities and GMH-IVH grade.^7^

MPV, determined by hematology analyzers, is a thrombocyte parameter measuring thrombocyte volume and activity. The presence of anisocytosis increases MPV, and increased MPV, which is a marker of thrombocyte production and consumption, can be associated with many diseases that can affect inflammation, hypoxia, and bone marrow 11. Our study results showed elevated MPV values in the preterms with GMH-IVH. In studies conducted on adults, there has been shown to be a relationship between mortality and the grade of diseases such as myocardial infarction, ischaemic stroke, mesenteric ischaemia, deep vein thrombosis, decompensated heart failure, subacute thyroiditis, and sepsis.^12^,^13^ In a study of children with Type-2 diabetes mellitus, the MPV value was found to be higher in the group with poor glycemic control. The authors associated this with the fact that larger platelets can produce denser prothrombotic factors and can thus adhere more easily.^14^ Different studies have shown a relationship with TTN, pneumonia, BPD, and sepsis, which may often be asociated with inflammation in the neonatal period.^15^,^16^ This could be attributed to both inflammations created by the existing condition and to platelet production and consumption caused by the hemorrhage. MPV shows platelet function and there is a positive correlation with aggregation and thromboxaneA2, platelet factor 4, and beta-thromboglobulin expression in thrombocytes. There are more dense granules in large volume platelets than in small thrombocytes and more serotonin and b-thromboglobulin are expressed. Increased MPV is associated with greater in-vitro aggregation as a response to adenosine diphosphate and collagen.^17^ Therefore, in the presence of GMH-IVH, which can develop with many different mechanisms in preterms, thrombocytes in the environment have a higher MPV because of turnover, inflammation, and aggregation.

Low-grade and high-grade hemorrhages were compared in this study, and the preterms with high-grade GMH-IVH were determined to have higher MPV values, although not at a statistically significant level. This was thought to be associated with the increased thrombocyte response. In previous studies conducted on adults, MPV has been shown to be associated with the presence and severity of disease and mortality.^16^,^17^ It has also been emphasized that elevated MPV in a group of newborns with necrotising enterocolitis was associated with disease severity and mortality.^18^ In some studies in neonates, MPV was not correlated with IVH, but was associated with platelet mass index and plateletcrit.^11^,^19^ In another study, similar to our study, MPV was found to be associated with preterm mortality but not with IVH.^20^ However, we demonstrated that increased MPV was associated with both IVH and antepartum mortality. To the best of our knowledge, this is the one of the few studies to have shown the relationship of increased MPV with both GMH-IVH and mortality. No relationship was determined between the MPV value and GA and birthweight, a cutoff value of >9.95 fL showed a probability of GMH-IVH in patients, and an MPV value of >10.55 fL could predict mortality.

### Limitations:

The major limitation of this study was the retrospective design, based on a single-centre cohort, although a highly reliable neonatal database that identified all the infants with GMH-IVH was able to be used. A second limitation could be said to be that MPV measurements were evaluated only once. Serial measurements may have been more reliable, but this was not possible because of the retrospective design as blood samples were only taken when required and not as routine in our unit.

### Strengths of study:

With regard to the strengths of our study, many factors that may have affected MPV were excluded, and the association between GMH-IVH and MPV as well as MPR could be evaluated more accurately. However, strong aspects could be said to be that the cranial ultrasonography imaging of all the cases was performed by the same two neonatologists. In addition, the application of similar routine care and protocols in a single centre was helpful in reducing confounding factors.

## CONCLUSION

In conclusion, increased MPV in GMH-IVH, which can easily develop for many multifactorial reasons in preterm infants who are extremely fragile, could be helpful in early diagnosis and be of guidance for clinicians. It has been shown that it can be of benefit in the early diagnosis of the presence of GMH-IVH and in the prediction of prognosis as a simple and low-cost marker in preterm infants. Following the conclusion of this study, we started to analyses the MPV values of neonates admitted to the NICU and to predict the risk of haemorrhage and mortality. In this way, we are being more cautious and keeping a closer eye on high-risk neonates. Nevertheless, there is a need for further, well-designed, prospective studies to be able to better define the role of MPV as a predictive marker in the clinical care of newborn infants. The serial measurement of MPV in these future studies will provide more robust evidence of the relationship between MPV and GMH-IVH.

### Author’s Contributions:

**IGV, AO, and GT:** Participated in the planning of this study.

**IGV and PD:** Participated in the data collection and reporting, reading.

**IGV and EDAC:** Data analysis. Critical Review.

All authors reviewed the results and approved the final version of the manuscript. They are also accountable for the integrity of the study.
